# Cell Electrosensitization Exists Only in Certain Electroporation Buffers

**DOI:** 10.1371/journal.pone.0159434

**Published:** 2016-07-25

**Authors:** Janja Dermol, Olga N. Pakhomova, Andrei G. Pakhomov, Damijan Miklavčič

**Affiliations:** 1 Faculty of Electrical Engineering, University of Ljubljana, Ljubljana, Slovenia; 2 Frank Reidy Research Center for Bioelectrics, Old Dominion University, Norfolk, Virginia, United States of America; Medical College of Georgia, UNITED STATES

## Abstract

Electroporation-induced cell sensitization was described as the occurrence of a delayed hypersensitivity to electric pulses caused by pretreating cells with electric pulses. It was achieved by increasing the duration of the electroporation treatment at the same cumulative energy input. It could be exploited in electroporation-based treatments such as electrochemotherapy and tissue ablation with irreversible electroporation. The mechanisms responsible for cell sensitization, however, have not yet been identified. We investigated cell sensitization dynamics in five different electroporation buffers. We split a pulse train into two trains varying the delay between them and measured the propidium uptake by fluorescence microscopy. By fitting the first-order model to the experimental results, we determined the uptake due to each train (i.e. the first and the second) and the corresponding resealing constant. Cell sensitization was observed in the growth medium but not in other tested buffers. The effect of pulse repetition frequency, cell size change, cytoskeleton disruption and calcium influx do not adequately explain cell sensitization. Based on our results, we can conclude that cell sensitization is a sum of several processes and is buffer dependent. Further research is needed to determine its generality and to identify underlying mechanisms.

## Introduction

Electroporation is a phenomenon resulting in a transient increase in membrane permeability, which occurs when short high voltage pulses are applied to cells and tissues [1,2]. If cells can recover, we consider this reversible electroporation. If the damage is too extensive and they die, we consider this irreversible electroporation (IRE). Electroporation is used in medicine, e.g. electrochemotherapy (ECT) [3–6], non-thermal IRE as a method of tissue ablation [7–9], gene therapy [10,11], DNA vaccination [12,13] and transdermal drug delivery [14–16], as well as in biotechnology [17], and food processing [18–20].

For tumor eradication, ECT, IRE, and gene therapy are successfully used. However, it was shown that electroporation of tumors larger than 2 cm in diameter is not as successful as of smaller tumors [21]. When treating tumors with IRE, a high number of pulses is delivered, which can cause significant Joule heating and thermal damage and complicate the treatment [22,23]. Provided the effect of the electric pulses be enhanced, we can treat larger tumors with fewer pulses of lower voltage. Pulse effect amplification can be achieved using molecules that enhance cell sensitivity to electric pulses, e.g. DMSO or surfactant C_12_E_8_ [8].

Lately, several reports on a so-called phenomenon of cell sensitization have been published [24–29]. By increasing the duration of an electroporation treatment (e.g. by decreasing the pulse repetition frequency or by splitting the delivered pulse train in more trains with delays between them), a decrease in cell viability and a much higher uptake of molecules were achieved. When applying conventional 100 μs pulses, 5 minute delay between the two trains was suggested [25], but also shorter delays led to cell sensitization [27,30]. Cell sensitization has been observed as decreased membrane integrity, increased mass transport across the membrane, and decreased cell viability. Similarly as with square pulses, it has been shown that exposing cells to AC electric fields increased their sensitivity to subsequent millisecond square pulses [31].

Cell sensitization could be beneficially used in the electroporation-based treatments. It is possible that it is already influencing the outcome of the IRE and the ECT. Namely, in the IRE, 90 pulses synchronized with a heartbeat are delivered between each pair of electrodes [8]. Usually, four electrodes are inserted, and IRE can last up to 9 minutes (four electrodes equals six pairs, 6x90 pulses at around 1 Hz take 9 minutes). In ECT, eight pulses at 1 Hz or 5 kHz are applied [32]. When using hexagonal electrodes, pulses are effectively delivered between 7 electrodes (12 pairs) [33]. Between each electrode pair, four pulses are delivered, and the procedure is repeated with four pulses of reverse polarity (twelve pairs, 8x12 pulses at around 1 Hz take 1.5 minute). If we consider the switching time [34], both treatments already fall within the time range for cell sensitization. The mechanisms of the delayed cell sensitization are not yet known. The proposed mechanisms are: 1) calcium uptake [24,25], 2) ATP leakage [24,25], 3) reactive oxygen species formation [24,25], 4) cell swelling [24,25], 5) cytoskeleton disruption [28], 6) reduced pore edge line tension which lowers the electroporation threshold [26,27], 7) extended pore opening times [26,27], and 8) the decrease of high conductance membrane state which allows for the creation of additional defects [35].

We would like to emphasize the difference in the definition of the cell sensitization in the already published studies and our paper. So far, cell sensitization has been defined as an increase in total molecular uptake or decrease in cell survival after applying a split dose as opposed to a single dose. The contribution of separate pulse trains to the final uptake and survival has not been investigated although, in our opinion, it is very important for the applicability of cell sensitization. When applying a single dose, we can reach saturation in mass transport [36,37]. Pulses at the end of the train contribute to the uptake less than pulses at the beginning [35,38]. With splitting the dose in half, we avoid saturation which is then mistakenly regarded as cell sensitization although cells respond to the first and the second pulse train in a similar way. In our experiments, we distinguished contributions of the first and the second pulse train. We considered the cells sensitized when the uptake due to the second pulse train was higher than the uptake due to the first train, irrespective of the final fluorescence level.

In preliminary experiments, we tested cell sensitization in a standard low-conductivity electroporation buffer. Surprisingly, splitting the dose in half lowered the final fluorescence. We were intrigued and decided to test propidium uptake in the growth medium, where cell sensitization has been previously observed [24,25,27]. There, splitting the dose in half increased the final fluorescence. We repeated experiments in three more electroporation buffers to investigate the effect of electrical conductivity, calcium influx, and sucrose concentration; splitting the dose in half again did not increase the final propidium uptake. Then, we analyzed the contributions of the separate pulse trains to the final fluorescence. The response of cells to pulse splitting in different electroporation buffers was complex and varied among others in the increased or decreased sensitivity to the second pulse train, in the resealing speed, and in the efficiency of split versus single dose. Although cell sensitization has already been defined as a phenomenon and models have been constructed describing it [39], further research is needed to determine its generality and the involved mechanisms.

## Materials and Methods

### Cell preparation

Chinese hamster ovary cells (European Collection of Authenticated Cell Cultures ECACC, cells CHO-K1, cat. no. 85051005, obtained directly from the repository) were grown in 25 cm^2^ culture flasks (TPP, Switzerland) for 2–3 days in an incubator (Kambič, Slovenia) at 37°C and humidified 5% CO_2_ in HAM-F12 growth medium (PAA, Austria). The growth medium was supplemented with 10% fetal bovine serum (Sigma-Aldrich, Germany), L-glutamine (StemCell, Canada) and antibiotics penicillin/streptomycin (PAA, Austria) and gentamycin (Sigma-Aldrich, Germany). On the day of the experiments, the cell suspension was prepared. Cells were detached by 2.5 ml of 10x trypsin-EDTA (PAA, Austria), diluted 1:9 in Hank’s basal salt solution (StemCell, Canada). After 1.5 minute the trypsin was inactivated by 2.5 ml of the growth medium. Cells were transferred to 50 ml centrifuge tube and centrifuged 5 minutes at 180g and 24°C. Then, cells were resuspended in the growth medium at 6x10^4^ cells/ml. 500 μl of cell suspension was added per well in 24-well plate. The plates were moved to the incubator for 15–40 minutes until cells attached to the bottom of the well but maintained their spherical shape [40].

Before the experiments, cells were washed with a fresh electroporation buffer and 500 μl of the new electroporation buffer was added to each well. For fluorescence measurements, the electroporation buffer included 150 μM propidium iodide (Life Technologies, USA). The composition of the tested electroporation buffers is given in Table 1. The electrical conductivity was measured with conductometers MA 5959 (Metrel, Slovenia) or S230 SevenCompact (Mettler Toledo, Switzerland) at 37°C and the osmolality by freezing point depression with Knauer cryoscopic unit (model 7312400000, Knauer, Germany). MgCl_2_, NaCl, K_2_HPO_4,_ CaCl_2_, HEPES, and sucrose were from Sigma-Aldrich, Germany, and KH_2_PO_4_ from Merck, Germany

**Table 1:** Composition of electroporation buffers.

Electroporation buffer	Composition	Electrical conductivity [mS/cm]	Osmolality [mOsm/kg]
The growth medium HAM-F12	Inorganic salts, amino acids, vitamins and other components (PAA Austria, cat. no. E15-016), 10% fetal bovine serum, L-glutamine, and antibiotics	17.14	260–320 (based on the producer’s data sheet)
The low-conductivity buffer	10 mM KH_2_PO_4_/K_2_HPO_4_ in ratio 40.5:9.5, 1 mM MgCl_2_, 250 mM sucrose	1.78	292
The hyperosmotic buffer	10 mM KH_2_PO_4_/K_2_HPO_4_, 1 mM MgCl_2_, 400 mM sucrose	1.76	475
The high-conductivity buffer	10 mM KH_2_PO_4_/K_2_HPO_4_ in ratio 40.5:9.5, 1 mM MgCl_2_, 150 mM NaCl	19.12	300
The buffer with calcium	10 mM HEPES, 250 mM sucrose, 0.7 mM MgCl_2_, 0.3 mM CaCl_2_	0.38	281

### Electroporation and image acquisition

**Fig 1:**
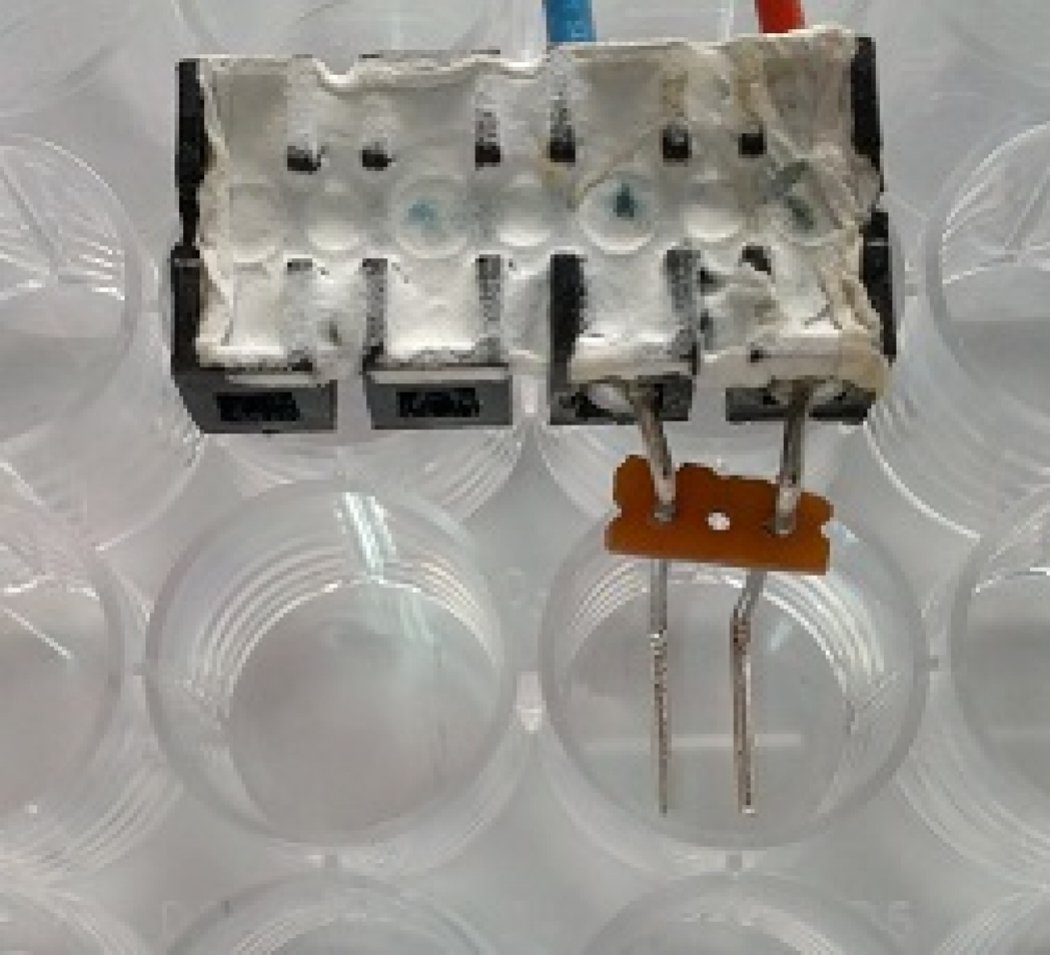
Photo of the electrodes attached to the bottom of the 24-well plate. The plate was put under a microscope. The images were acquired from between the electrodes where the electric field was approximately homogeneous and could be calculated as the ratio of the applied voltage and inter-electrode distance.

**Fig 2:**
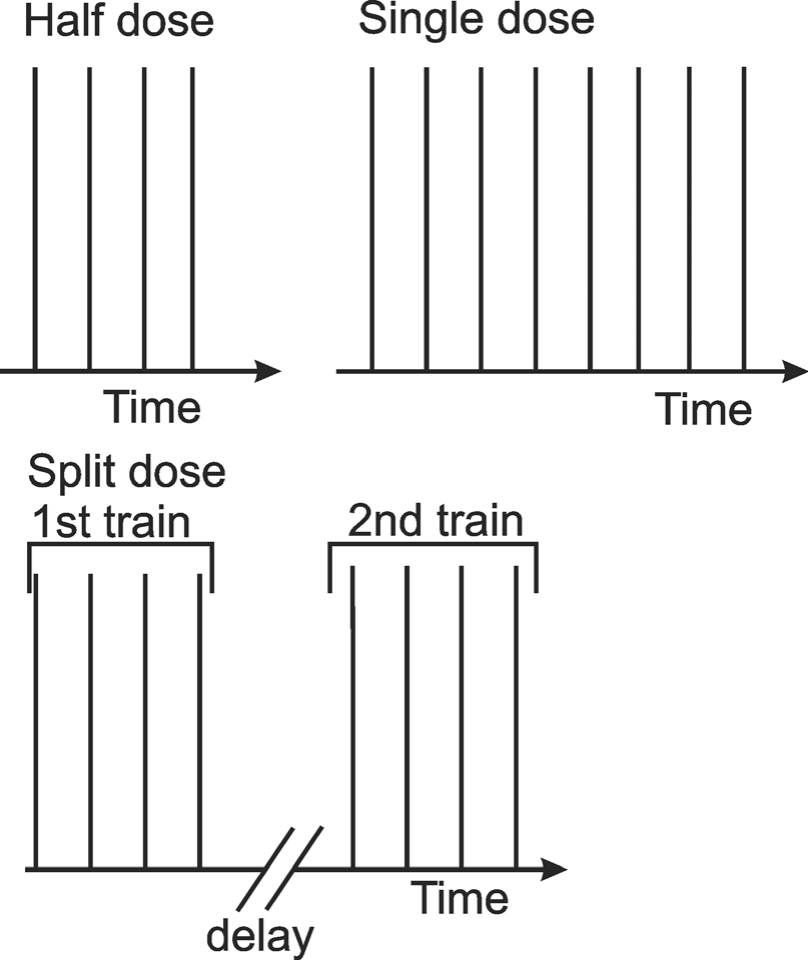
Scheme of the used protocols. In the half dose, four 100 μs pulses, and in the single dose, eight 100 μs pulses were applied. In the split dose, two trains of four 100 μs pulses were applied and the delay between the end of the first and the beginning of the second train was 1, 2 or 3 min. In all experiments, 300 V or 0.75 kV/cm were applied. The repetition frequency was 10 Hz.

We used Pt/Ir wire electrodes with 0.8 mm diameter and 4 mm inter-electrode distance positioned at the bottom of the plate as shown in Fig 1. The plate was put under the microscope with a heated stage (37°C). Square pulses (300V or 0.75 kV/cm, 100 μs, 10 Hz) were applied using the βtech electroporator (Electro cell B10, Betatech, France) and monitored with an oscilloscope WaveSurfer 422, 200 MHz and a current probe AP015 (Teledyne LeCroy, Chestnut Ridge, NY). In fluorescence measurements, we exposed cells to either half dose (four 100 μs pulses), single dose (eight 100 μs pulses) or split dose (4+4, 100 μs pulses with 1, 2 or 3 minute delay) (Fig 2). In the cell size measurements, we exposed cells only to the half dose (four 100 μs pulses). 300 V was chosen based on the preliminary experiments, where most of the cells were permeabilized, but the survival was not affected. 3 minutes was chosen as the delay that produced cell sensitization in other studies [30] and where the measured final fluorescence value of the split dose was higher (the growth medium) or lower (the low-conductivity buffer) than of the single dose.

Cells were observed by the inverted microscope AxioVert 200 (Zeiss, Germany) under 20x objective for fluorescence (535 nm excitation, 617 nm emission) and 40x for cell size measurements. Images were acquired using the VisiCam 1280 camera (Visitron, Germany) and the MetaMorph PC software (Molecular Devices, USA) in a time lapse: one image every 8 or 10 s for 8 minutes (fluorescence measurements) or 2 s for 3 minutes (cell size measurements). We chose a field of view in the middle between the electrodes where the electric field distribution was approximately homogeneous, and electric field was calculated as a ratio of the applied voltage to inter-electrode distance [41]. The numbers of the experiments and the analyzed cells for each protocol are shown in Table 2. The exposure time in fluorescence measurements was different across tested buffers, and the absolute values of fluorescence should not be directly compared.

**Table 2:** Number of experiments and analyzed cells in each experiment.

Type of experiment			No. of experiments	No. of analyzed cells
Fluorescence measurement	The growth medium	4 pulses	3	19
		8 pulses	5	34
		4+4 pulses, 1 min delay	6	31
		4+4 pulses, 2 min delay	6	37
		4+4 pulses, 3 min delay	7	40
	The low-conductivity buffer	4 pulses	5	34
		8 pulses	6	45
		4+4 pulses, 1 min delay	6	38
		4+4 pulses, 2 min delay	8	55
		4+4 pulses, 3 min delay	7	48
	The hyperosmotic buffer	4 pulses	4	21
		8 pulses	3	20
		4+4 pulses, 3 min delay	4	23
	The high-conductivity buffer	8 pulses	3	24
		4+4 pulses, 3 min delay	3	23
	The buffer with calcium	8 pulses	3	21
		4+4 pulses, 3 min delay	3	25
Cell size measurement	The growth medium		4	19
	The low-conductivity buffer		7	25

Images were analyzed using the ImageJ software (http://imagej.nih.gov/ij/). On fluorescent images, each cell was manually outlined, and its average fluorescence intensity through the whole stack was automatically measured. Fluorescence intensity before the pulse application was subtracted to compensate for the background fluorescence. In preliminary experiments, it was determined that in all tested buffers, there was no measurable spontaneous propidium uptake on the same time scale without applying electric pulses. For cell size measurements, the threshold was applied to the bright-field images, cells were automatically outlined, and their area in each image was measured. The measured area was normalized to the size of the area before pulse application to determine a relative change in the cross-section. In each time step, the average value and the standard error were determined.

### The model fitting

The quantitative results of the uptake were acquired by fitting a first-order model [42]:

$$f(t)=C(1-\exp\left(-\frac{t}{\tau }\right))$$ (1)

to the experimental results using the Curve fitting toolbox and Matlab R2011b (Mathworks, USA). *S* signifies the plateau of the fluorescence; *τ* is the relaxation time of propidium uptake when the cells reach 63% of the final fluorescence. The time course of propidium uptake reflects the resealing process of the membrane and *τ* can also be understood as a resealing constant [42]. The derivation is in the S1 Appendix.

**Fig 3:**
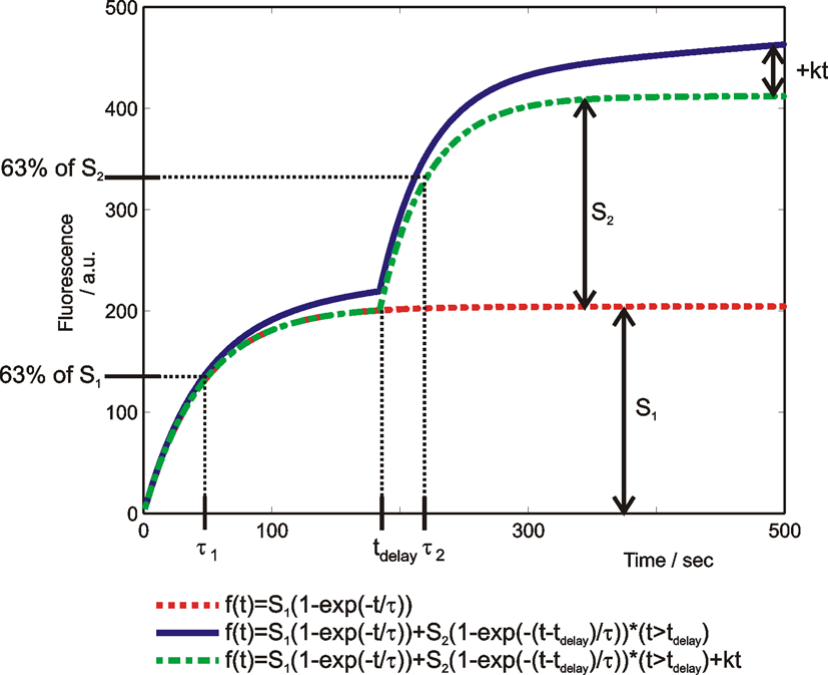
An example of fitted first-order models to the results of propidium uptake in the growth medium. Meaning of *S*_*1*_, *S*_*2*_, *τ*_*1*_ *τ*_*2*_, *t*_*delay*_ and the increase due to ‘*kt*’ are shown.

The model:

$$f(t)=S_{1}(1-exp\left(-\frac{t}{\tau_{1}}\right))+S_{2}(1-exp\left(-\frac{t-t_{delay}}{\tau_{2}}\right))*(t>t_{delay})+kt$$ (2)

was fitted to the split dose results. Parameter *t*_*delay*_ is the inter-train delay, *k* is the slope of the linear part of the propidium uptake. An example of the fitted curve and the parameters is shown in Fig 3. The first order kinetics model described the majority of our data sufficiently well (*R*^*2*^>0.97), but it predicted that after the exponential closing of the pores the transport will stop, and the fluorescence will not increase. In our experimental results, the shape of the propidium uptake curves indicated that there was an additional process present that caused the linear uptake. We modeled it with a linear uptake process (‘*kt*’) which we assumed present from the beginning of the treatment. Since we do not know what exactly caused the linear uptake, it seemed inappropriate to ascribe it only to one of the trains.

We fitted Eq 1 to the half and the single dose and Eq 2 to the split dose results. The optimal values of the parameters and the *R*^*2*^ (the coefficient of determination) value are presented in Table 3. In the split dose protocols, we normalized the *S*_*2*_ and *τ*_*2*_ values to the average *S*_*1*_ or *τ*_*1*_ value in the same buffer (unless stated differently in Table 3). If *S*_*2*_/*S*_*1*_>1, cells were assumed sensitized, if *S*_*2*_/*S*_*1*_<1, they were assumed desensitized, and if *S*_*2*_/*S*_*1*_≈1 the trains were assumed equally effective. Ratio *τ*_*2*_/*τ*_*1*_ gave information whether the resealing after the second train was faster (*τ*_*2*_/*τ*_*1*_<1) or slower (*τ*_*2*_/*τ*_*1*_>1) than after the first train or similar to the first train (*τ*_*2*_/*τ*_*1*_≈1). The values of parameters *S*_*1*_ and *τ*_*1*_ should be the same for all first trains (half and split dose) in each buffer. The difference in their values was due to the biological variability and the error of curve fitting. The error of curve fitting was more noticeable if the delay between two pulse trains was short and the first-order shape of the uptake curve was not very clear. Therefore, in order to decrease the error we presented results by normalizing the *S*_*2*_ and *τ*_*2*_ to the average values of *S*_*1*_ and *τ*_*1*_ for each buffer separately. In the cell (de)sensitization definition, ‘*kt*’ was not included, since we assumed this additional process present from the beginning of the measurement.

**Table 3:** The optimal values of parameters and the *R*^*2*^ of fitted Eqs 1 and 2. A–The growth medium, B–the low-conductivity buffer, C–the hyperosmotic buffer, D–the high-conductivity buffer, E–the buffer with calcium. Values in bold and the symbol next to them emphasize if the cells were desensitized (*S*_*2*_*/average(S*_*1*_*)*<1, symbol **↓**), sensitized (*S*_*2*_*/average(S*_*1*_*)*>1, symbol **↑**) or if the trains were equally effective (*S*_*2*_*/average(S*_*1*_*)*≈1, symbol **≈**). The numbers after ± sign define 95% confidence interval. The graphical representation of *S*_*2*_*/average(S*_*1*_*)* and *τ*_*2*_*/average(τ*_*1*_*)*, based on experimental uptake results in Fig 4, is in Fig 5.

**A–The growth medium**					
	Half dose	Single dose	1 min delay	2 min delay	3 min delay
*S*_*1*_	215.5 ± 7.5	354.8 ± 9.9	179.6 ± 14.2	197.4 ± 17.2	204.2 ± 8.2
*S*_*2*_	/	/	265.9± 13.9	226.9 ± 12.5	207.6 ± 8.8
*τ*_*1*_	65.91 ± 4.17	63.45± 3.33	44.02± 6.16	69.47± 10.07	46.13 ± 3.44
*τ*_*2*_	/	/	48.37 ± 1.58	47.01± 3.25	37.76 ± 2.98
*k*	0.06664 ± 0.02	0.06825 ± 0.0267	0.1831 ± 0.0124	0.1234 ± 0.0339	0.1024 ± 0.0372
***S***_***2***_***/average(S***_***1***_***)***	/	/	**1.34 ± 0.18 ↑**	**1.14 ± 0.15↑**	**1.04 ± 0.13≈**
*τ*_*2*_*/average(τ*_*1*_*)*	^/^	/	0.86 ± 0.20	0.83 ± 0.20	0.67 ± 0.16
*R*^*2*^	0.9954	0.9961	0.9998	0.9993	0.9991
**B–The low-conductivity buffer**					
	Half dose	Single dose	1 min delay	2 min delay	3 min delay
*S*_*1*_	516.9 ± 3.9	965.7 ± 1.7	516.9 ± 3.9^a^	516.9 ± 3.9^a^	445.4 ± 19.4
*S*_*2*_	/	/	413.1 ± 4.9	269 ± 12.4	229.2 ± 10.7
*τ*_*1*_	91.6 ± 2.66	82.78 ± 0.6	91.6 ± 2.66^a^	91.6 ± 2.66^a^	86.7 ± 4.97
*τ*_*2*_	/	/	51.58 ± 3.15	65.11 ± 3.40	44.95 ± 3.65
*k*	0	0	0	0.0985 ± 0.0293	0.1512 ± 0.0556
***S***_***2***_***/average(S***_***1***_***)***	/	/	**0.86 ± 0.04 ↓**	**0.56 ± 0.03↓**	**0.48 ± 0.03↓**
*τ*_*2*_*/average(τ*_*1*_*)*	/	/	0.58 ± 0.05	0.73 ± 0.06	0.50 ± 0.05
*R*^*2*^	0.9962	0.9997	0.9980	0.9994	0.9996
**C–The hyperosmotic buffer**					
	Half dose	Single dose	3 min delay		
*S*_*1*_	191 ± 2.4	387.7 ± 4.5	155.8 ± 27.3		
*S*_*2*_	/	/	86.76 ± 10.28		
*τ*_*1*_	161.1 ± 5.3	117.8 ± 4.5	124.8 ± 21.2		
*τ*_*2*_	/	/	69.63 ± 12.46		
*k*	0	0	0.01741 ± 0.0662		
***S***_***2***_***/average(S***_***1***_***)***	/	/	**0.50 ± 0.08 ↓**		
*τ*_*2*_*/average(τ*_*1*_*)*	/	/	0.49 ± 0.10		
*R*^*2*^	0.9977	0.9958	0.9992		
**D–The high-conductivity buffer**					
	Single dose		3 min delay		
*S*_*1*_	681.2 ± 9.1		369.8 ± 31.4		
*S*_*2*_	/		379.8 ± 8.6		
*τ*_*1*_	57.54 ± 1.54		122.2 ± 11.7		
*τ*_*2*_	/		36.68 ± 1.34		
*k*	0.4366 ± 0.0255		0.2719 ± 0.0623		
***S***_***2***_***/average(S***_***1***_***)***	/		**1.03 ± 0.09 ≈**		
*τ*_*2*_*/average(τ*_*1*_*)*	/		0.30 ± 0.10		
*R*^*2*^	0.9992		0.9999		
**E–The buffer with calcium**					
	Single dose^b^		3 min delay		
*S*_*1*_	1129 ± 432		365.6 ± 22.4		
*S*_*2*_	/		386.1 ± 22		
*τ*_*1*_	144 ± 55.9		79.36 ± 11		
*τ*_*2*_	/		69.86 ± 5.54		
*k*	0		0		
***S***_***2***_***/average(S***_***1***_***)***	/		**1.06 ± 0.88 ≈**		
*τ*_*2*_*/average(τ*_*1*_*)*	/		0.88 ± 0.14		
*R*^*2*^	0.9705		0.9974		

^a^ The values for the *S*_*1*_ and *τ*_*1*_ were taken from the results of fitting Eq 2 to the results of the half dose (2^nd^ column) where the plateau of fluorescence was already reached, and the first order shape was clear.

^b^ Here, the first order model was not appropriate since the 95% confidence interval was very large. These parameters do not influence the results of the analysis since the ratios were not calculated from the single dose parameters.

## Results

**Fig 4:**
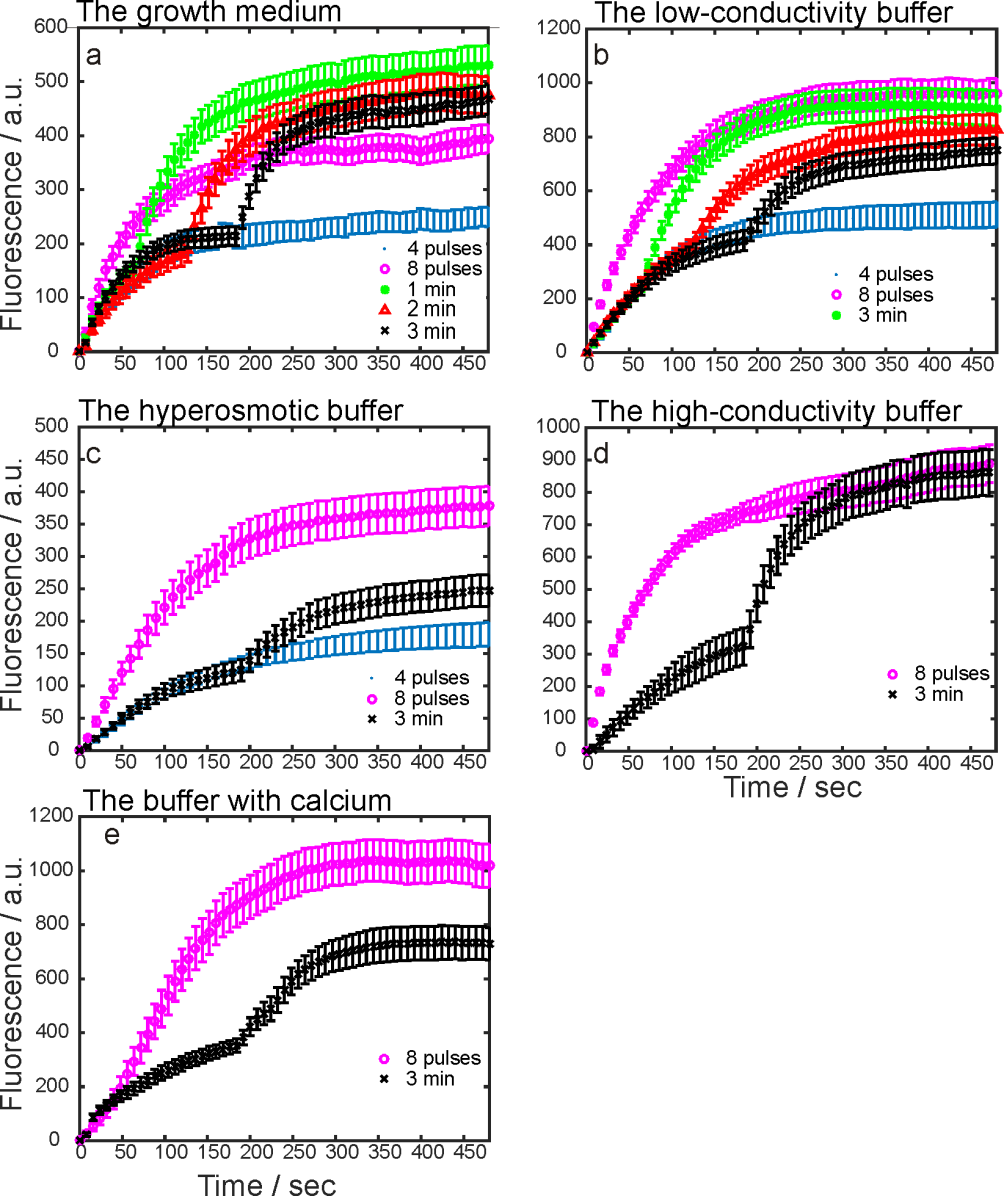
Measured fluorescence due to the propidium uptake in the growth medium and four different electroporation buffers. Propidium uptake was detected by fluorescence microscopy. On the *y*-axis, there are the average experimental values in arbitrary units ± standard error. On the *x*-axis, there is the time in seconds. (a), the growth medium; (b), the low-conductivity; (c) the hyperosmotic; (d), the high-conductivity buffer, (e) the buffer with calcium. The exposure times were different among the electroporation buffers.

**Fig 5:**
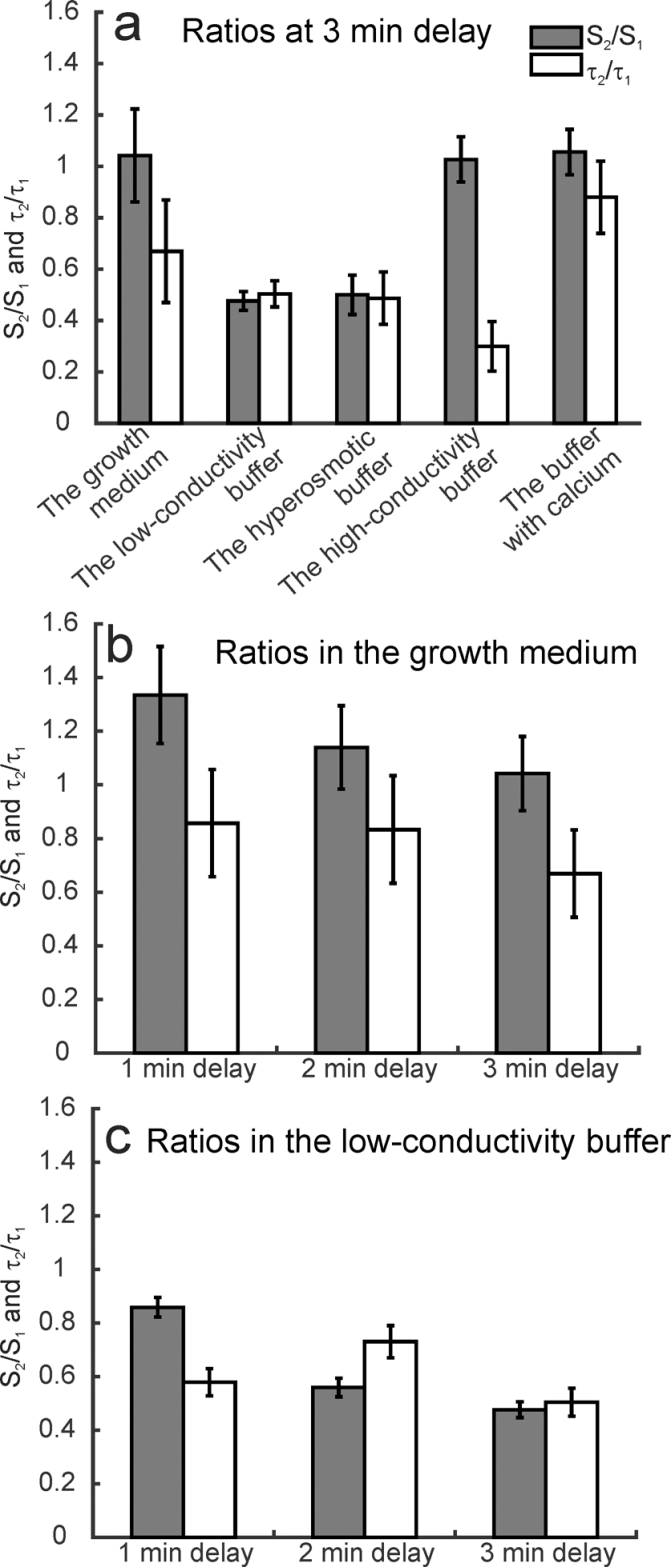
Graphical representation of the ratio of parameters *S*_*2*_*/S*_*1*_ and *τ*_*2*_/*τ*_*1*._ A first order model (Eqs 1 and 2) was fitted to the experimental results. *S*_*2*_ and *τ*_*2*_ were normalized to the average value of *S*_*1*_ or *τ*_*1*_, calculated from parameters of the half dose and the split doses with different delays. (a) The ratios at 3 minute delay between the trains in all buffers. (b) The ratios in the growth medium at 1, 2 or 3 minute delay. (c) The ratios in the low-conductivity buffer at 1, 2 and 3 minute delay. The bars at 3 minute delay in (b) and (c) represent the same results as the growth medium and the low-conductivity buffer bars in (c) to enable easier comparison of the ratios between the buffers. Error bars represent the 95% confidence interval.

First, experiments were performed with a fixed inter-train delay (3 min) in five electroporation buffers. Results are presented in Cell sensitization in different buffers section. Second, different inter-train delays were tested in the growth medium and the low-conductivity buffer. These two buffers were chosen as a representative for the cell sensitization and cell desensitization effect. Results are presented in Cell sensitization with different delays section. The time dynamics of propidium uptake in the growth medium, the low-conductivity, the hyperosmotic, the high-conductivity buffer, and the buffer with calcium are shown in Fig 4A, 4B, 4C, 4D and 4E, respectively. The observations on higher or lower uptake as a response to the first or second train are based on fitting Eqs 1 and 2 to the experimental results. One of the fitted curves is plotted in Fig 3 where the parameters *S*_*1*_, *S*_*2*_, *t*_*delay*_, *τ*_*1*,_ and *τ*_*2*_ are shown. In Table 3, the optimal values of parameters and the calculated ratios of *S*_*2*_/*S*_*1*_ and *τ*_*2*_/*τ*_*1*_ are presented. If *S*_*2*_/*S*_*1*_>1, cells were assumed sensitized, if *S*_*2*_/*S*_*1*_<1, they were assumed desensitized, and if *S*_*2*_/*S*_*1*_≈1 the trains were assumed equally effective. The final level of fluorescence was irrelevant in our definition of cell sensitization. In Fig 5A, there is the graphical representation of the ratios in the growth medium, the low-conductivity, the hyperosmotic, the high-conductivity buffer, and the buffer with calcium at fixed 3 minute inter-train delay. In Fig 5B, ratios for the growth medium, and in Fig 5C for the low-conductivity buffer at different delays are shown.

### Cell sensitization in different buffers

In the growth medium, the total uptake after a split dose protocol (i.e. 4+4 pulses) was higher than after a single dose protocol (i.e. eight pulses) at 1 minute delay (Fig 4A). The second train contributed more to the total fluorescence (Fig 5A), i.e. cell sensitization was present. The value of *k* was similar for the single and the half dose, but it was much higher for the split dose. There was a gradual uptake of propidium even 8 minutes after the pulses (*k* > 0).

In the low-conductivity buffer, the split dose protocol resulted in lower final fluorescence than the single dose protocol (Fig 4B). In the split dose protocol, the uptake due to the second train was lower than due to the first train (Fig 5A), i.e. cell sensitization was not present. On the contrary, cell desensitization was present. The value of *k* was 0 for single, half dose and 1 minute delay.

In the hyperosmotic buffer, the cells were exposed to half, single and split dose with 3 minute delay (Fig 4C). The split dose was less effective than the single dose. The uptake due to the second pulse train was lower than the uptake due to the first train (Fig 5C), i.e. cell desensitization was present. The parameter *k* was more than 0 only in the split dose protocol, and it was 10-times smaller than in the low-conductivity buffer (the same exposure time allows us to make the comparison).

In the high-conductivity buffer, the cells were exposed to the single and split dose with 3 minute delay (Fig 4D). The uptake due to the second train was similar to the uptake due to the first train (Fig 5C), i.e. cell sensitization was not present. The uptake due to the split dose was twice the uptake due to the half dose (determined by extrapolation). The *k* after the single dose was higher than after the split dose.

In the buffer with added calcium, the final uptake due to the single dose was higher than due to the split dose (Fig 4E). However, the uptake due to the second train was similar to the uptake due to the first train, i.e. cell sensitization was not present (Fig 5A). The value of *k* was 0 for the split and single dose.

The values of *τ* after the second pulse train were lower than after the first pulse train in the growth medium and all electroporation buffers, i.e. the resealing after the second pulse train was faster than after the first train (Table 3). The values of *τ* after the first train were the lowest in the growth medium (44–69 s^-1^) and then they increased in the order: the buffer with added calcium (69–79 s^-1^), the low-conductivity (86–91 s^-1^), the high-conductivity (122 s^-1^), and the hyperosmotic buffer (124–161 s^-1^).

**Fig 6:**
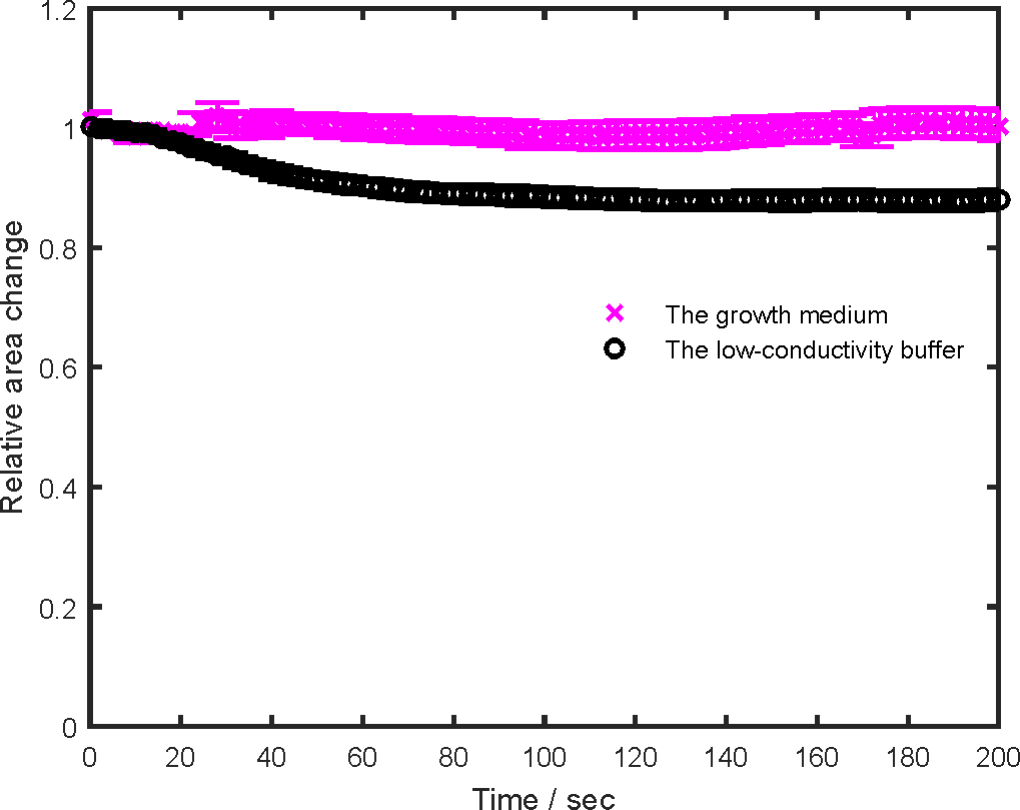
Cell size change dynamics in the growth medium and the low-conductivity buffer. Symbols denote the average ± standard error. The relative change was determined as a ratio of the cell cross-section after and before the pulses. The upper magenta curve–the growth medium; the lower black curve–the low-conductivity buffer.

In the growth medium and the low-conductivity buffer, we also determined the cell size change after four 100 μs pulses (the half dose) (Fig 6). Cell size change was proposed as a possible mechanism of cell sensitization [24]. In the growth medium, the cell cross-section did not change much. In the low-conductivity buffer, it decreased. In the low-conductivity buffer, the dynamics of cell size change was different from the dynamics of the fluorescence change due to the second train (Fig 4C). The uptake due to the second train was lower at 3 than at 2 minute delay while the cell cross-section did not change from the first minute until the end of the measurement.

### Cell sensitization with different delays

In the growth medium and the low-conductivity buffer, additional experiments with 1 and 2 minutes delay between the trains were performed (Fig 4A and 4B). In the growth medium, the cell sensitization decreased with increasing inter-train delay (Fig 5B). At 3 minute delay, there was no cell sensitization; the fluorescence was a sum of the separate contributions of the pulse trains. The parameter *k* decreased with an increasing inter-train delay as well. In the low-conductivity buffer, we observed cell ‘desensitization’ (*S*_*2*_/*S*_*1*_<1) which increased with increasing delay. The value of *k* was zero for single, half dose, and 1 minute delay.

## Discussion

Cell sensitization was not present in all tested electroporation buffers as determined by the propidium uptake measurements. In the growth medium, the split dose was more efficient than the single dose at all delays, but the cells were sensitized by the first train only at 1 min. In other buffers, the split dose was similarly (the high-conductivity buffer, and the buffer with calcium), or less effective (the low-conductivity, and the hyperosmotic buffer) than the single dose. In these buffers, thus cell sensitization was not present. After pulse application, different processes are triggered in the cells or on their membranes. These processes apparently depend also on the electroporation buffer composition and influence the response of the cells to the electric pulses. The sum of these processes determines whether the cells will be sensitized, desensitized or the trains will be equally effective. In our study, if *S*_*2*_/*S*_*1*_>1, cells were assumed sensitized, if *S*_*2*_/*S*_*1*_<1, they were assumed desensitized, and if *S*_*2*_/*S*_*1*_≈1 the trains were assumed equally effective. Cell sensitization, assessed by the uptake of different molecules or by survival after a single or a split dose protocol, was observed when applying pulses in the growth medium [24,25,28,30], in electroporation buffers [24,25,43], in three-dimensional cell cultures [29], and *in vivo* [26,27]. Our results in the growth medium are in agreement with them, but the results in the other buffers are not.

With our definition of cell sensitization (higher uptake due to the second than due to the first pulse train), the sensitizing effect was observed only at 1 minute delay in the growth medium (Fig 5B). However, the final level of fluorescence in the growth medium was still higher when applying the split dose than when the single dose with all delays tested (1, 2 or 3 min). In previously published studies, the higher final fluorescence of the split than of the single dose was already considered cell sensitization. There, cells were assumed sensitized if the split dose caused higher uptake or lower survival than the single dose. With their definition, in our experiments in the growth medium, all delays would be considered as cell sensitizing although the second pulse train did not cause higher uptake than the first pulse train, i.e. the cells were not sensitized during the inter-train delay.

### Pulse splitting in different buffers

In the growth medium, cell sensitization was present, and the split dose was more effective (Figs 4A and 5A) than the single dose. The reason could be higher membrane damage due to the first pulse train than in other tested buffers which rendered cells more sensitive to the subsequent pulses. With increasing delay, the damage was repaired, and cell sensitization ceased. In other studies [24–27,30], the cell sensitization was present at longer delays. The possible explanations are: 1) They assessed cell sensitization as the efficiency of the split *vs* single dose. In our experiments, the final value of the fluorescence was higher when applying the split dose, although separate contributions to the final fluorescence of the two trains were equal. 2) They damaged the membrane more by applying more pulses of higher electric field. Since higher electric fields cause higher cell sensitization [30], they could also cause longer cell sensitization. 3) Our experiments were performed at 37°C where the membrane resealing is faster [44] while their experiments were performed at room temperature.

In the low-conductivity buffer, there were protecting processes happening which take more than 3 minutes to finish since the effect of ‘desensitization’ was still present with 3 minutes delay. The time dynamics of propidium uptake was different than in the growth medium which indicates that different mechanisms are involved. In the hyperosmotic buffer, the second train of the split dose was even less effective than in the low-conductivity buffer. Since one of the differences between these buffers was sucrose concentration, we can assume that it has an important role, as discussed in the following paragraphs. In the high-conductivity buffer, split and single dose caused similar final fluorescence (Fig 4G). Although sucrose inhibited cell sensitization, its absence did not cause it.

Time constant *τ* describes the time dynamics of the membrane resealing. Membrane resealing is affected by several parameters. It was slower when the membrane was oxidized [45] and faster at higher temperatures [44], lower sucrose concentration [46], and in the presence of low Ca^2+^ concentrations [42]. The cell membrane resealing depended on calcium, which had to be present in low concentrations when the cells were damaged either chemically, mechanically or electrically [42,47–49]. The increased intracellular calcium concentration after electroporation served as a stress indicator that triggered the membrane resealing process in the form of endo- [49], and exocytosis [47,48]. In the growth medium and the buffer with calcium, there were low concentrations of Ca^2+^ present and *τ* was lower than in other buffers (faster resealing), probably due to Ca^2+^ facilitated membrane resealing. Even when calcium was not added, it was still present during electroporation. First, calcium is present in the intracellular storages [50]. Second, even if it is not added to the buffer, there is some free calcium which comes from the glass or impurities in the chemicals, as measured in [45] for the low-conductivity buffer. Interestingly, in [47], the resealing after a second membrane disruption was much faster than after the first one. Authors suggested that initial wound was healed by exocytosis of the vesicles from the endocytic compartment while the second wound resealed faster since Golgi apparatus (responsible for a formation of new vesicles) had already been activated by an increased Ca^2+^ influx. The role of Golgi apparatus is in agreement with our results since the resealing constant *τ* after the second pulse train was lower (faster resealing) than after the first one in all electroporation buffers (Table 3). Parameter *τ* decreased with increasing delay since there was more time for the activation of the Golgi apparatus. The resealing speed after the half and the single dose was very similar in all tested buffers, contrary to what was observed by Rols and Teissié [51]. However, they applied more pulses (10 or 20 as opposed to 4 or 8 in our study) of higher intensity (1.2 kV/cm as opposed to 0.75 kV/cm in our study) which caused more pronounced membrane damage and difference in the resealing speed.

Parameter *k* describes the additional uptake process on a longer time scale. A higher value of *k* indicates either higher final fluorescence or faster resealing since $k=\frac{S}{\tau }$, as derived in the S1 Appendix. Thus, *k* depends on the final fluorescence and inversely depends of the resealing time constant. In the growth medium, this additional process was always present. The value of *k* decreased with an increasing delay, which suggests that either 1) the fluorescence due to the additional process was decreasing or 2) its resealing speed was increasing. The decrease in the final fluorescence could be caused by the decreasing membrane permeabilization between the trains. The second train caused less damage, and the additional uptake was lower than at shorter delays. In the low-conductivity buffer, the additional process of linear propidium uptake was not always present. The trend of *k* was different than in the growth medium. With increasing delay, the final fluorescence of the additional process was increasing since slower resealing is not likely (longer delay should decrease the resealing time). When the additional process was not present, it finished too fast or was too small to be observed. With longer delays, the additional process was visible because it was not finished yet. Parameter *k* does not reflect the binding delay of propidium after it enters the cells since it binds in microseconds [52].

In [43] pulses were split into two trains, and propidium or Yo-Pro-1 uptakes were measured. Interestingly, under the same pulse parameters, there was a large cell sensitization effect present with propidium, but much smaller with Yo-Pro-1 [53]. Apparently, different dyes give different uptake results under the same conditions. Although pulses in the nanosecond range were applied, the observation is interesting also for the microsecond pulses since there are reports on cell sensitization with nanosecond [24] as well as with microsecond [25–27,30] pulses.

### Possible mechanisms of cell (de)sensitization

Increasing sucrose concentration caused among others an increase in osmotic pressure which decreased the size or density of the permeabilizing structures in the membrane, slower membrane resealing [46] and stabilization of cell membrane [54]. Similarly, trehalose (a disaccharide similar to the sucrose) had a protective and stabilizing role [55,56]. Our results are in agreement with slower membrane resealing observation since *τ* increased with increasing sucrose concentration. Higher sucrose concentration also caused lower fluorescence due to the second pulse train—the cells were protected by a still unknown mechanism which is also in agreement with the protective role of the sucrose. When the sucrose was replaced by NaCl, the pulse trains were equally effective. Sucrose inhibits cell sensitization, but its absence does not facilitate it.

The electrical conductivity of the growth medium and the high-conductivity buffer was in the same range. In the high-conductivity buffer, there was no cell sensitization, while in the growth medium, cell sensitization was observed. Since a similar electrical conductivity induces a different cell response, it is considered not to be responsible for cell sensitization.

In [35], authors ascribed cell sensitization to the higher efficiency of lower pulse repetition frequencies. Applying pulses on a permeabilized cell membrane is less effective because the existing conducting structures prevent the establishment of an additional transmembrane potential, which they call ‘cell desensitization'. (The expression was used in a different sense than in our paper.) Assuming that cell sensitization is a consequence of repetition frequency effect, one cannot explain such different behaviors in different buffers. Their explanation of cell sensitization effect is thus unlikely.

Extended pore opening times have also been proposed among possible mechanisms [26,27]. The duration of all the protocols was the same; the membrane resealing was in the same time range. Thus, the pore opening times were also of approximately the same duration. The extended pore opening time does not explain cell sensitization.

Cell size change after electroporation occurs due to the colloid osmotic pressure [57,58]. Electric pulses of different parameters cause different extent of cell permeability, and the cell membrane becomes permeable to small molecules but not to large ones. The colloid osmotic pressure drives the influx of water and small solutes in the cell, and the cell swells [2]. Molecules, larger than the pores, balance colloid osmolality and stop cell swelling [58]. Increased/decreased cell size could cause higher/lower induced transmembrane voltage according to the Schwann equation. Cell size was measured via visible cross-section. In the growth medium, the cell size did not increase and thus cannot be the reason for cell sensitization. In the low-conductivity buffer, the cells shrunk. Cell desensitization could in part be explained by cell shrinking and lower induced transmembrane voltage. However, cell shrinking cannot be the only reason since the time dynamics of the uptake due to the second train and the cross-section change were different.

Cell sensitization has been observed in several cell lines and *in vivo*. In our experiments, we used Chinese hamster ovary cells. Since in this cell line cell sensitization either was [25] or was not present, it does not depend only on the cell line. It is possible that even in the growth medium cell sensitization would not be present in certain cell lines; however this remains to be further investigated.

The cytoskeleton is damaged by electric pulses [59–61] and there were different observations made (e.g. damaged cytoskeleton does not influence electroporation [62,63], it increases permeabilization [28], it increases the survival [62], it changes the resealing dynamics [61]). Cytoskeleton disruption has also been connected to cell swelling [60]. In [28], the disruption of the cytoskeleton rendered cells more sensitive to electric pulses. However, the repair of the cytoskeleton is in the range of hours [59], but the cell sensitization dynamics in our experiments was in the range of minutes. The cytoskeleton disruption is not a likely explanation for cell sensitization.

The concentration of calcium we used in our buffer with calcium was similar as in the HAM-F12 growth medium. In the buffer with calcium, cells were more sensitive to the second pulse train than in the low-conductivity buffer. However, this was due to the lack of sucrose in the buffer with calcium. In the high-conductivity buffer where there is no calcium, the ratio *S*_*2*_*/S*_*1*_ was 1.03 and in the buffer with calcium, it was 1.06. In both buffers, the first and the second pulse train were equally effective. It is possible that higher calcium concentrations would cause cell sensitization but they could also lower cell viability [64]. Namely, electroporation with 1 mM calcium already decreased survival of various cell lines to 20–40% [65].

In the literature, also other explanations for cell sensitization were suggested: ATP leakage, membrane oxidation, and reduced membrane line tension. The investigation of these factors is beyond the scope of our current study. Our study opens many more questions which deserve being investigated in future: the mechanism of sucrose’s protection of the membrane, the effect of different pulse parameters (duration and the number of pulses, electric field).

Modeling of cell electroporation is very useful since it allows us to describe cell responses, make predictions about effects of electric pulses on the cells, decrease the number of experiments needed, and help us to understand what is happening [37,39,66–68]. However, until also biological effects are included in the models, they will have a limited power and will not correctly predict the outcome in certain cases.

Cell sensitization has also been observed *in vivo* by the delayed tumor growth, enhancement of tumor necrosis and perfusion defects [26,27]. The growth medium is an approximation of the *in vivo* extracellular fluid. The mechanism(s) for cell sensitization *in vitro* and *in vi*vo could be similar. There are, however, many more factors *in vivo*–cell crowding, blood supply, immune system, electric field shielding.

## Conclusions

Cell sensitization seems to be a buffer dependent phenomenon. In the growth medium, still unknown processes were triggered by the pulse application and rendered cells more susceptible to electric pulses. In the low-conductivity and hyperosmotic buffer, a protective effect of the sucrose and cell shrinking caused cell ‘desensitization’. In the high-conductivity buffer, there was no cell ‘desensitization’, probably due to the lack of sucrose. There was also no cell sensitization present since the cell ‘sensitizing’ processes from the growth medium were missing. The exact mechanism of cell sensitization has still to be determined; however we believe we shed additional light on the existing hypotheses and discarded some of them. The effect of pulse repetition frequency [35], cell size change, cytoskeleton disruption [24] and calcium influx [24] seem unlikely explanations. Based on our results, there are different sensitizing and desensitizing mechanisms present and competing at the same time, and the outcome depends on their overall contributions.

## Supporting Information

S1 Appendix Derivation of the first-order uptake equation. (DOCX)
