# Supplementary material for: Cell Electrosensitization Exists Only in Certain Electroporation Buffers
Source: PLoS One. 2016 Jul 25;11(7):e0159434. doi: 10.1371/journal.pone.0159434 (PMC4959715; doi:10.1371/journal.pone.0159434)
Supplement: S1 Appendix — (DOCX) [file pone.0159434.s001.docx]

**S1 Appendix: Derivation of the first-order uptake equation**

We can follow a similar derivation as [1] and assume that after a pulse application cell permeabilization decreases exponentially with time:

| $P(t)=P_{0}\exp\left( -\frac{t}{\tau} \right),$ | (1) |
| --- | --- |

where *P(t)* means the permeability at time *t*, *P_0_* is the initial permeability at the end of the pulse and *τ* is the resealing constant. Flux through the electroporated membrane can be written as in [2]:

| $j(t)=P_{0}\exp\left( -\frac{t}{\tau} \right)\left( c_{e}-c_{i} \right)\approx P_{0}\exp\left( -\frac{t}{\tau} \right)c_{e},$ | (2) |
| --- | --- |

where c*_i_* means the internal and c*_e_* the external concentration. We assume that all propidium in the cell binds to nucleic acids immediately and that the intracellular propidium concentration is 0 mM. Namely, in [3] it has been shown that the propidium signal starts to increase microseconds after the pulse start. In our experiments, we measured the uptake in the range of seconds, and we could neglect the time delay of propidium binding. Since in our experiments, we did not reach propidium saturation (the fluorescence value of triton treated cells was below the maximal measured fluorescence of electroporated cells) we can assume that there was no unbound propidium in cells and thus the internal propidium concentration was 0 mM. The number of molecules *N* that enter the cells can be obtained by integration of *j* over time and permeabilized area ([2], Equation 9):

| $N\left( t \right)=P_{0}\tau c_{e}AN_{A}(1-exp \left( -\frac{t}{\tau} \right)),$ | (3) |
| --- | --- |

where *A* is the permeabilized area and *N_A_* the Avogadro constant. By uniting *τ*, *c_e_*, *P_0_*, *A* and *N_A_* into a constant *C* we obtain a first-order model:

| $N(t)=C\left( 1-\exp\left( -\frac{t}{\tau} \right) \right).$ | (4) |
| --- | --- |

The fluorescence of propidium is linearly dependent on the number of the bound molecules below the saturation level [4]. The constant *C* does not have any meaning; it is only a multiplicative factor and describes the plateau of the reached fluorescence. Thus, the fluorescence can be written as:

| $f(t)=S(1-\exp\left( -\frac{t}{\tau} \right))$, | (5) |
| --- | --- |

where *f* signifies the fluorescence in dependence on time and *S* is constant. The expression (5) can be directly applied to our measurements.

The shape of the propidium uptake curves indicated that there is an additional process present that causes the linear uptake seen after the first order process is finished. If we assume that this process is also first-order and that its time constant is much larger than our observation time, we can use linear expansion and include the first two terms:

| $\exp\left( t \right)=1+\frac{t}{1!}+\frac{t^{2}}{2!}+\ldots=\sum_{n=0}^{\infty} \frac{t^{n}}{n!}.$ | (6) |
| --- | --- |

We obtain an approximation of Equation (5):

| $f(t)=S\left( 1-\exp\left( -\frac{t}{\tau} \right) \right)\cong\frac{S}{\tau}t=kt,$ | (7) |
| --- | --- |

where *k* equals the plateau of the process divided by the resealing constant.

1. Shirakashi R, Köstner CM, Müller KJ, Kürschner M, Zimmermann U, Sukhorukov VL. Intracellular Delivery of Trehalose into Mammalian Cells by Electropermeabilization. J Membr Biol. 2002;189: 45–54. doi:10.1007/s00232-002-1003-y

2. Miklavčič D, Towhidi L. Numerical study of the electroporation pulse shape effect on molecular uptake of biological cells. Radiol Oncol. 2010;44. doi:10.2478/v10019-010-0002-3

3. Pucihar G, Kotnik T, Miklavčič D, Teissié J. Kinetics of Transmembrane Transport of Small Molecules into Electropermeabilized Cells. Biophys J. 2008;95: 2837–2848. doi:10.1529/biophysj.108.135541

4. Kennedy SM, Ji Z, Hedstrom JC, Booske JH, Hagness SC. Quantification of electroporative uptake kinetics and electric field heterogeneity effects in cells. Biophys J. 2008;94: 5018–5027. doi:10.1529/biophysj.106.103218
